# Anesthesia advanced circulatory life support

**DOI:** 10.1007/s12630-012-9699-3

**Published:** 2012-04-21

**Authors:** Vivek K. Moitra, Andrea Gabrielli, Gerald A. Maccioli, Michael F. O’Connor

**Affiliations:** 1 Department of Anesthesiology, College of Physicians and Surgeons, Columbia University, New York, NY USA; 2 Anesthesia Department, University of Florida, Gainesville, FL USA; 3 American Anesthesiology of North Carolina/MEDNAX, Raleigh, NC USA; 4 Department of Anesthesia and Critical Care, University of Chicago, 5841 S Maryland Ave, MC 4028, Chicago, IL 60637 USA

## Abstract

**Purpose:**

The constellation of advanced cardiac life support (ACLS) events, such as gas embolism, local anesthetic overdose, and spinal bradycardia, in the perioperative setting differs from events in the pre-hospital arena. As a result, modification of traditional ACLS protocols allows for more specific etiology-based resuscitation.

**Principal findings:**

Perioperative arrests are both uncommon and heterogeneous and have not been described or studied to the same extent as cardiac arrest in the community. These crises are usually witnessed, frequently anticipated, and involve a rescuer physician with knowledge of the patient’s comorbidities and coexisting anesthetic or surgically related pathophysiology. When the health care provider identifies the probable cause of arrest, the practitioner has the ability to initiate medical management rapidly.

**Conclusions:**

Recommendations for management must be predicated on expert opinion and physiological understanding rather than on the standards currently being used in the generation of ACLS protocols in the community. Adapting ACLS algorithms and considering the differential diagnoses of these perioperative events may prevent cardiac arrest.

## Résumé

**Objectif:**

Le grand ensemble d’événements liés à la réanimation cardiaque avancée (ACLS) tels que les embolies gazeuses, les surdosages d’anesthésiques locaux et la bradycardie sinusale dans un contexte périopératoire est différent des événements que l’on observe à l’extérieur de l’hopital. En conséquence, une modification des protocoles traditionnels d’ACLS permet une réanimation plus spécifique, en fonction de l’étiologie.

**Constatations principales:**

Les arrêts cardiaques en période périopératoire sont à la fois rares et hétérogènes; ils n’ont pas été décrits ou étudiés avec la même ampleur que les arrêts cardiaques survenant hors de l’hôpital. Ces crises sont habituellement vécues en direct, souvent anticipées et impliquent l’intervention d’un médecin connaissant les comorbidités du patient ainsi que la physiopathologie en rapport avec l’intervention et les anesthésiques utilisés. Lorsque le professionnel de la santé identifie la cause probable de l’arrêt cardiaque, le praticien a la possibilité d’entreprendre rapidement une prise en charge médicale.

**Conclusions:**

Des recommandations pour la prise en charge doivent être fondées sur les avis d’experts et sur la compréhension de la physiologie plutôt que sur des normes actuellement utilisées pour la création de protocoles d’ACLS hors du milieu hospitalier. L’adaptation des algorithmes d’ACLS et la prise en compte des diagnostics différentiels de ces événements périopératoires peuvent prévenir les arrêts cardiaques.

## Part 1

### Introduction

Advanced cardiac life support (ACLS) was originally developed as an extension of basic life support with a focus on the resuscitation of individuals found unresponsive in the community by providing chest compression and respiratory support. These clinical interventions were later expanded to immediate care in the emergency department and were then exported to unresponsive patients elsewhere in the hospital.

The initiation of cardiopulmonary resuscitation (CPR) is predicated on the discovery of an unresponsive patient who does not have a pulse.^1^ Advanced cardiac life support is rhythm oriented and specific to sudden manifestations of cardiac and respiratory diseases outside of the hospital. This approach presumes that effective chest compression, adequate ventilation, and electrical and pharmacological management of a pulseless cardiac rhythm will result in the return of spontaneous circulation (ROSC).^2^,^3^

Cardiac arrest during anesthesia is distinct from cardiac arrest in other settings because it is usually witnessed and frequently anticipated. Compared with other settings, the response may be both more timely and focused. In the perioperative setting, a patient with a known medical history typically deteriorates into crisis over a period of minutes or hours under circumstances wholly dissimilar to other in-hospital or out-of-hospital scenarios. Consequently, aggressive measures can be taken to support the patient’s physiology and avoid or delay the need for ACLS. Additionally, patients in the perioperative period have a different pathophysiologic milieu. For example, hypovolemia is far more common than a transmural myocardial infarction from plaque rupture. Similarly, prolonged hypoxemia and hypercarbia resulting from the management of unpredictable difficult airways is a well-recognized cause of cardiac arrest in the operating room (OR).^4^-^7^ Bradycardiac arrest in the OR is caused or precipitated by vagotonic analgesics, physical manipulations that increase vagal tone, and sympatholysis from anesthetic agents and regional/neuraxial anesthetic techniques.^8^,^9^

A large prospective and retrospective case analysis study of all perioperative cardiac arrests occurring during a ten-year period (1989-1999) in a single teaching institution showed an overall incidence of cardiac arrest from all causes of 19.7 per 10,000 anesthetics and a risk of death related to anesthesia-attributable perioperative cardiac arrest of 0.55 per 10,000 anesthetics.^4^ In a review of cardiac arrests associated with anesthesia, the most common electrocardiogram (ECG) rhythms at the time of arrest were bradycardia (23%), asystole (22%), tachydysrhythmia (including ventricular tachycardia and ventricular fibrillation) (14%), and normal (7%). Remarkably, in 33% of the cases, the heart rhythm was not fully assessed or documented.^10^ The introduction of worldwide safety standards and improved understanding of the physiologic impact of anesthetic agents is consistent with recent epidemiological data suggesting an improvement of anesthesia-related mortality risk in the United States and abroad, with the highest death rate found in very elderly patients (≥ 85 yr).^11^

Although the cause of circulatory arrest is usually unknown in patients found in the field, there is a relatively short list of probable causes of circulatory collapse during the perioperative period.^4^-^6^,^8^,^9^ This certainty produces more focused and etiology-based resuscitation efforts, which frequently do not follow the more generic algorithms of the ACLS guidelines.

### Anesthesia and cardiac arrest

Common situations associated with perioperative cardiac arrest are listed in Table 1. A comprehensive list of trends in anesthesia-related deaths is available from an analysis of insurance-based claims covering 13,000 anesthesiologists in North America.^7^ Herein, several common scenarios are analyzed in detail.

**Table 1:** Common situations associated with perioperative cardiac arrest

**Anesthetic**
Intravenous anesthetic overdose
Inhalation anesthetic overdose
Neuraxial block with high level sympathectomy
Local anesthetic systemic toxicity
Malignant hyperthermia
Drug administration errors
**Respiratory**
Hypoxemia
Auto-PEEP
Acute bronchospasm
**Cardiovascular**
Vasovagal reflex
Hypovolemic and/or hemorrhagic shock
Surgical maneuvers associated with reduced organ blood flow
Gas embolism
Acute electrolyte imbalance (high K, low Ca^++^)
Increased intra-abdominal pressure
Transfusion reaction
Anaphylactic reaction
Tension pneumothorax
Acute coronary syndrome
Pulmonary thromboembolism
Severe pulmonary hypertension
Pacemaker failure
Prolonged Q-T syndrome
Oculocardiac reflexes
Electroconvulsive therapy

PEEP = positive end-expiratory pressure

#### Neuraxial anesthesia

Epidemiology reviews of cardiac arrest during neuraxial anesthesia suggest an incidence of 1.3-18 per 10,000 patients.^9^,^12^-^15^ A recent review of cardiac arrests during neuraxial anesthesia reports a prevalence of 1.8 cardiac arrests per 10,000 patients, with more arrests occurring in patients receiving spinal anesthesia *vs* all the other techniques (2.9 *vs* 0.9 per 10,000 patients, respectively; *P* = 0.041). In this review, cardiac arrest during neuraxial anesthesia was associated with a greater likelihood of survival compared with cardiac arrest during general anesthesia.^9^

Although there is a substantial amount of basic science and clinical interest in the effects of high spinal anesthesia on the sympathetic innervation of the heart and circulation,^16^-^22^ the pathophysiology of cardiac arrest remains unclear. Various hypotheses have been proposed, invoking factors such as unrecognized respiratory depression, excessive sedation concurrent with a high block, underappreciation of both the direct and indirect circulatory consequences of a high spinal anesthetic, and failure to rescue with airway management and drugs.^8^,^9^,^23^-^26^ Hypoxemia from hypoventilation is an unlikely etiology because there are case reports documenting adequate oxygen saturation in these patients. Neuraxial cardiac arrests are most likely precipitated by the combination of autonomic imbalance with enhanced vagal tone and an acute decrease in preload from venodilatation.^8^,^9^

During the perioperative period, cardiac arrest in association with neuraxial anesthesia is difficult to predict^8^,^9^,^27^ and may occur more than 40 min after injection.^9^,^12^ Proposed risk factors for bradycardia or cardiac arrest during spinal anesthesia include American Society of Anesthesiologists (ASA) physical status I *vs* ASA physical status III or IV, age < 50 yr, heart rate < 60 beats·min^−1^, prolonged P-R interval, use of beta-blocking drugs, and a sensory level above T6.^8^ In a review of neuraxial cardiac arrests, almost 50% of cardiac arrests were associated with recurrent specific surgical events (cementing of joint components, spermatic cord manipulation, manipulation of a broken femur, blood loss, and rupture of amniotic membranes).^9^ Avoiding anesthesia during surgical manipulation is not feasible, making it difficult to determine the contribution of anesthesia and manipulation in isolation to the development of neuroaxial cardiac arrest. The pathophysiology of this phenomenon is unclear, and the selection of vasoactive drugs during neuraxial anesthesia is controversial.^28^ A proposed treatment protocol is outlined in Table 2.

**Table 2:** Treatment of cardiac arrest associated with neuraxial anesthesia

**Pre-Arrest**
• Discontinue anesthetic or sedation infusion
• Immediate tracheal intubation and ventilation with 100% oxygen
• Treat bradycardia with 1 mg atropine
• Treat bradycardia with severe hypotension with *at least* 1 mg epinephrine *iv*
• Consider transcutaneous or intravenous pacemakers for all symptomatic bradycardic rhythms with pulse
• Consider chest compressions at a rate of 100 compressions·min^−1^ if above measures are ineffective
**Cardiac Arrest**
• Immediate CPR as indicated (no carotid pulse, absence of ECG rhythm, loss of arterial catheter, and pulse oximeter signal)
• Epinephrine 1 mg *iv*; consider alternative approach to drug therapy, i.e., escalating doses or reducing epinephrine time interval to every 1–2 min
• Consider concurrent treatment with vasopressin 40 U *iv*

CPR = cardiopulmonary resuscitation; ECG = echocardiogram

#### Local anesthetics

Although the risk of local anesthetic toxicity is difficult to predict, the risk for toxicity increases with dose and site of injection.^29^ In general, local anesthetics depress the heart in a dose-dependent fashion and can cause bradycardia, asystole, decreased contractility, and hypotension.^29^-^31^ Other determinant factors of anesthetic blood level and systemic toxicity are the site of injection and the rate of absorption. Direct intravascular injection of a local anesthetic typically produces an immediate toxic effect, whereas toxicity from absorption over time from well-vascularized peripheral tissue, such as the pleural space, may present in a delayed fashion.^29^

Bupivacaine, the local anesthetic agent most often associated with cardiac arrest, is a potent and well-described myocardial depressant.^30^-^34^ Fortunately, most awake patients who are developing systemic toxicity manifest early neurological symptoms that may suggest impending myocardial dysfunction. In some unfortunate patients, however, these changes presage cardiac arrest.

Manifestations of local anesthetic toxicity include ringing in the ears or buzzing in the ears, metallic taste or perioral tingling, dysphasia, orthostatic hypotension, confusion, premature ventricular contractions, wide QRS complex ECG (which can subsequently deteriorate into pulseless electrical activity [PEA] or asystole [bupivacaine]), sinus bradycardia or atrioventricular block (lidocaine and etidocaine), and decreased myocardial contractility. A treatment protocol for this complication is given in Table 3.^35^-^41^

**Table 3:** Treatment of local anesthetic toxicity

**Pre-Arrest**
• Stop the administration of local anesthetic
• Immediate tracheal intubation and ventilation with 100% oxygen
• Consider transcutaneous or intravenous pacemakers for all symptomatic bradycardic rhythms with pulse
• If the diagnosis of local anesthetic toxicity is strongly suspected, the use of epinephrine should be avoided as it can worsen outcome^35^,^36^
• Consider chest compressions at rate of 100 compressions·min^−1^ if above measures are ineffective
• 20% intralipid *iv*, 1.5 mL·kg^−1^ *iv* load, then 0.25 mL·kg^−1^·hr^−1^ 37,38 (the efficacy of this therapy is still debated and under investigation, and it should complement and not substitute the immediate use of catecholamines^39^,^40^)
• Seizures should be treated with benzodiazepines. Small doses of propofol or thiopental may be used if benzodiazepines are not immediately available^41^
**Cardiac Arrest**
• Immediate CPR as indicated (no carotid pulse, ECG, arterial catheter, and pulse oximeter signal)
• If the diagnosis of local anesthetic toxicity is strongly suspected, small doses of epinephrine 10-100 μg are preferable to higher doses^41^
• Vasopressin is not recommended^41^
• Sodium bicarbonate to maintain a pH > 7.25 in patients without immediate ROSC after CPR and drug therapy
• If ROSC does not occur following the first bolus of lipid emulsion, a second bolus followed by a doubling of the rate of infusion is appropriate^41^
• Consider therapy with H1 and H2 blockers
• Amiodarone is the drug of choice for ventricular arrhythmias. Lidocaine should be avoided^41^
• Most important, continue CPR for a prolonged period (we suggest at least 60 minutes) as very good neurologic recovery has been reported in patients after very prolonged cardiac arrests from local anesthetic overdoses
• ECMO may be appropriate in circumstances where the diagnosis is certain, where ECMO is available in a timely fashion, and where there is no ROSC after a second bolus of lipid emulsion^41^

CPR = cardiopulmonary resuscitation; ECG = electrocardiogram; ROSC = return of spontaneous circulation; ECMO = extracorporeal membrane oxygenation

#### Anaphylaxis

Anaphylaxis is a rare but important cause of circulatory collapse in the perioperative period.^27^,^42^ The reported incidence of anaphylaxis is from one in 10,000 to one in 20,000 anesthetics, with 3-10% of those cases being life-threatening.^43^-^45^ Although there is a wide range of minor allergic reactions, hypotension, tachycardia, bronchospasm, and vasoplegic shock may follow when the offending agent is administered as a rapid intravenous bolus - the most common route of drug administration during anesthesia.^46^ The preponderance of anaphylaxis in perioperative patients is caused by a small number of drugs, particularly neuromuscular blocking agents.^43^,^47^

Common causes of anaphylactic shock include nondepolarizing neuromuscular blockers, beta-lactam antibiotics, latex exposure, and intravenous contrast.

The focus for management of a patient with anaphylaxis is on interrupting the reaction and supporting the patient (Table 4).^48^,^49^ If feasible, surgery should be interrupted, and the patient should be immediately supported with intravenous fluids and vasopressors.^34^ Early consideration should be given to securing the airway if the patient experiences inspiratory stridor or develops facial and lip swelling. It is imperative to remember that epinephrine administered to patients with anaphylaxis is intended to both interrupt the reaction and support circulation.^50^ Thus, it should be given at an initial bolus dose and followed by an epinephrine infusion titrated to maintain a systolic blood pressure of at least 90 mmHg. Adjunct therapies, such as arginine vasopressin, corticosteroids, and H1 and H2 receptor antagonists, may be considered and are listed in Table 4.^42^,^48^

**Table 4:** Treatment of anaphylaxis

**Pre-Arrest**
• Stop or remove the inciting agent or drug (e.g., intravenous contrast or latex)
• If feasible, stop surgery or procedure
• Oxygen at FIO_2_ of 1.0; intubate immediately for respiratory distress
• Epinephrine 0.5-3 μg·kg^−1^; start epinephrine infusion (5-15 μg·min^−1^) for a goal SBP 90 mmHg; observe for myocardial ischemia
• Watch for auto-PEEP if severe bronchospasm
• ± Vasopressin 2 U *iv*
• Intravenous fluids/large bore access
• H1 blocker (diphenhydramine 50 mg *iv*)
• H2 blocker (famotidine 20 mg *iv*)
• ± Corticosteroid (e.g., 50-150 mg hydrocortisone *iv*)
• A tryptase level in the blood can be used to support the diagnosis^48^
**Cardiac Arrest**
• CPR if no carotid pulse detected for 10 sec
• Epinephrine 1 mg *iv*, can repeat every 3-5 min or follow by vasopressin 40 U
• Disconnect the ventilator briefly if auto-PEEP suspected
• Consider tension pneumothorax if arrest preceded by severe bronchospasm
• Add adjunctive therapies listed in pre-arrest

FIO_2_ = fraction of inspired oxygen concentration; SBP = systolic blood pressure; PEEP = positive end-expiratory pressure; CPR = cardiopulmonary resuscitation

#### Gas embolism

Gas embolism is an important cause of circulatory crisis and cardiac arrest in perioperative patients. As the number of procedures using minimally invasive techniques involving gas insufflation increases, the frequency of intraoperative gas embolisms will likely increase. The risk for a venous air embolism increases when the surgical field is above the right atrium, particularly in patients with low central venous pressure (CVP). The focus of hemodynamic support is on improving right ventricular (RV) function^51^ (Table 5).

**Table 5:** Treatment of gas embolism

**Pre-Arrest**
• Administer 100% oxygen and intubate for significant respiratory distress or refractory hypoxemia. Oxygen may reduce bubble size by increasing the gradient for nitrogen to diffuse out
• Promptly place patient in Trendelenburg (head down) position and rotate toward the left lateral decubitus position. This maneuver helps trap air in the apex of the ventricle, prevents its ejection into the pulmonary arterial system, and maintains right ventricular output
• Maintain systemic arterial pressure with fluid resuscitation and vasopressors/beta-adrenergic agents if necessary. See the algorithm for RV failure
• Consider transfer to a hyperbaric chamber if immediately available. Potential benefits of this therapy include compression of existing air bubbles, establishment of a high diffusion gradient to speed dissolution of existing bubbles, improved oxygenation of ischemic tissues, and lowered intracranial pressure
**Cardiac Arrest**
• Circulatory collapse should be addressed with CPR, and consideration should be given to more invasive procedures as described above
• Early use of TEE to rule out other treatable causes of pulmonary embolism
• Consider the right ventricular shock algorithm

RV = right ventricular; CPR = cardiopulmonary resuscitation; TEE = transesophageal echocardiography

Common causes of gas embolism include laparoscopy, endobronchial laser procedures, central venous catheterization or catheter removal, hysteroscopy, pressurized wound irrigation, prone spinal surgery, posterior fossa surgery in the sitting position, and pressurized fluid infusion.

Massive gas embolisms have been characterized by breathlessness, continuous coughing, arrhythmias, myocardial ischemia, acute hypotension with loss of end-tidal carbon dioxide, and cardiac arrest.^51^

#### Acute hyperkalemia

Hyperkalemia can be an elusive but important cause of cardiac arrest in perioperative patients regardless of pre-existing kidney injury. It is important for practitioners to appreciate that many patients who sustain a hyperkalemic cardiac arrest do not appear to undergo the orderly deterioration of cardiac rhythm (peaked T waves followed by a widened QRS complex and eventually the classic sine wave) that has been widely taught. Electrocardiographic changes may be absent in hyperkalemia.^52^ Life-threatening hyperkalemia (> 6.5 mmol·L^−1^) can slow atrioventricular conduction or cause asystole, ventricular tachycardia, ventricular fibrillation, and PEA. Several congenital or acquired conditions, including burns and upper/lower motor neuron lesions, can cause upregulation of nicotinic acetylcholine receptors and cardiac arrest via a massive release of potassium, especially when intravenous succinylcholine is used to facilitate intubation.^53^ The prevalence of hyperkalemia as a cause of cardiac arrest in hospitalized patients is sufficiently high that it should be included in the differential diagnosis of every patient with a new wide-complex arrhythmia. Its treatment has been widely described in the literature.^49^

#### Malignant hyperthermia

Malignant hyperthermia is a rare but potentially fatal manifestation of hypermetabolism which is triggered by exposure to specific drugs in susceptible individuals. The hallmark of this rare syndrome is a sudden and rapid increase of uncompensated oxygen consumption in individuals exposed to succinylcholine and volatile inhalation agents, including enflurane, halothane, isoflurane, sevoflurane, and desflurane. Inherited genetic mutations of the sarcoplasmic reticulum can result in massive release of intracellular calcium after exposure to a triggering agent.^54^-^56^ Clinical features of malignant hyperthermia include unexplained tachycardia with hypertension, rapid increase in end-tidal CO_2_ without hypoventilation, increased minute ventilation if the patient is breathing spontaneously, uncompensated or mixed metabolic acidosis (lactic) with increased PaCO_2_, a rapid or delayed (up to a few hours) increase in temperature, hyperkalemia from rhabdomyolysis with severe dysrhythmias, myoglobinuria, disseminated intravascular coagulation (DIC) and “nonsurgical” bleeding, localized masseter muscle or generalized muscle rigidity, and massive increase of plasma creatine kinase. A clinician-based diagnosis of DIC substantially increases the risk of cardiac arrest and death.^57^ The differential diagnosis for malignant hyperthermia includes sepsis, thyrotoxicosis, pheochromocytoma, iatrogenic warming, CO_2_ rebreathing, and neuroleptic malignant syndrome.

Mortality from malignant hyperthermia was as high as 70% before the widespread awareness of this complication, its early recognition, and its timely treatment with intravenous dantrolene.^54^ The mortality from malignant hyperthermia has decreased, and a recent study of patients in the United States reports a mortality rate of 11.7%.^58^ Malignant hyperthermia with cardiac arrest carries a mortality of 50% in healthy young individuals under anesthesia.^57^ The most effective way to prevent malignant hyperthermia is to avoid use of triggering agents in patients suspected or known to be susceptible.^55^

A treatment protocol for malignant hyperthermia is given in Table 6.

**Table 6:** Treatment of malignant hyperthermia

• Discontinue all anesthetic and switch from the anesthesia ventilator to manual Ambu bag ventilation from a separate source of oxygen. Switch to a dedicated *clean* anesthesia ventilator or transport or ICU ventilator when feasible. Continue E_T_CO_2_ monitoring if feasible
• Stop surgery when feasible
• Switch to intravenous anesthetic if necessary
• Sodium dantrolene (be sure you know where it is in your hospital and how to prepare it): give 2.5 mg·kg^−1^ or 1 mg·lb^−1^ initial dose. Repeat bolus of Na dantrolene, titrating to tachycardia and hypercarbia (10 mg·kg^−1^ suggested upper limit, but more may be given as needed, up to 30 mg·kg^−1^)
• Begin active cooling: ice packs to groin, axilla, and neck; cold intravenous solutions into the peritoneal cavity when feasible; nasogastric or peritoneal lavage when feasible
• Stop cooling measures at 38°C to avoid overshooting
• If hyperkalemia suspected by peaked ECG T waves or intraventricular conduction delay confirmed by high K serum level: calcium chloride 10 mg·kg^−1^, insulin 0.1 U·kg^−1^ + 50 mL D50w for adult or 1 mL·kg^−1^ for pediatrics. Repeat as necessary
• Metabolic acidosis: 100 mEq of HCO_3_^−^ in adults, then titrate to pH 7.2. Normalize pH if confirmed rhabdomyolysis (suggested threshold, CPK 10,000 IU·L^−1^)
• Respiratory acidosis: treatment is controversial due to adverse hemodynamic effects of hyperventilation if low-flow state is confirmed. (We suggest an initial goal of modest permissive hypercarbia with a goal E_T_CO_2_ of 50-60 mmHg)
• Dysrhythmias: avoid calcium antagonists after Na dantrolene, potential for worsening hyperkalemia
• Myoglobinuria with oliguria: place Foley catheter; increase rate of fluid resuscitation
• Invasive pressure monitoring when feasible, more HCO_3_^−^ to neutralize urine pH, consider intravenous mannitol
• Supportive measures for disseminated intravascular coagulation (DIC)
• Call for help, including the MH hotline, if feasible (www.mhaus.org) call 1-800-644-9737 or 1-800-MH-HYPER in the USA and Canada; outside the USA, call 00113144647079
• When the crisis is resolved: Consider caffeine-halothane muscle biopsy *in vitro* contracture test, molecular genetic testing for genetic mutation analysis for patient’s relatives (sensitivity 25%)

Table adapted from http://www.mhaus.org/ website

ICU = intensive care unit; E_T_CO_2_ = end-tidal carbon dioxide; ECG = electrocardiogram; D50W = dextrose in water (50%); CPK = creatine phosphokinase; MH = malignant hyperthermia

#### Complications of central venous access

In the category of equipment-related damaging events, the ASA Closed Claims Project found that central venous placement is the most frequent event associated with death and permanent brain damage during anesthesia.^7^ Although a pneumothorax is a well-described and relatively rare complication of central venous catheter placement or removal, an analysis from the ASA closed-claims database suggests that hemothorax and tamponade are also important though sometimes unrecognized fatal complications of patients who undergo attempts at central venous cannulation.^58^,^59^ If a patient’s hemodynamic situation deteriorates following central venous catheter placement, expeditious focused echocardiography should be considered in addition to chest radiography.^60^

## Part 2

### Introduction

When ACLS was first introduced, it was the consensus product of a multidisciplinary group with a common interest in ACLS. At the time, there was little scientific evidence to guide and shape the guidelines that the group eventually authored. Fortunately, there was an interest in scenarios that were sufficiently common to permit systematic study, which facilitated subsequent revisions of the ACLS guidelines.^61^ However, given that cardiac arrest occurs rarely in the perioperative period, it is difficult, or impossible, to perform large epidemiological studies and generate evidence-based guidelines.

Recent surveys among anesthesiologists suggest a lack of awareness of both basic and anesthesia-related knowledge regarding resuscitation from cardiac arrest.^62^,^63^ One recent study has documented delay in the cardioversion and defibrillation of patients with shockable rhythms in the perioperative setting*.* 64 Despite these challenges, detailed reviews of this topic are now available,^65^ and there is a wealth of expertise and experience among anesthesiologists in managing both circulatory crisis and cardiac arrest in perioperative patients.

After performing a review of the relevant literature, we offer these suggestions, hoping that they will inspire systematic studies and more formal guidelines to manage these rare perioperative events.

### Pre-cardiac arrest issues

Rescuing a patient from an intraoperative crisis requires two separate and very distinct components: *comprehension* that the patient is in crisis and *effective action.* 66-68 Some clinicians may not recognize the early signs and symptoms of physiological deterioration that often precede adverse events.^66^ *Failure to rescue* may be a misidentified “cause” of cardiac arrest. Regrettably, in some instances, failure to rescue is really the inability to rescue a patient from an underlying process that becomes so severe (after delayed recognition of a crisis in evolution) that an adverse event, including death, is inevitable despite the timely institution of maximal support.^66^,^69^

#### Escalating care

In addition to initiating therapies during crisis, it is also appropriate to consider escalating the level of monitoring to correspond with the level of supportive care. Clinicians should assess the patient’s known comorbidities, surgical logistics, hemodynamic effects of the anesthetics used, and ongoing autonomic nervous system modulation (e.g., tachydysrhythmias with hypotension *vs* bradydysrhythmias with hypotension). The timely insertion of both an arterial catheter and a central venous catheter will likely be very helpful in the serial evaluation and management of patients (outlined below).^70^ Insertion of invasive monitors should not take precedence over supportive measures. The decision to escalate the level of monitoring, similar to the decision to escalate therapies, is ultimately a clinical decision that considers a large number of patient and surgical factors and is beyond the scope of these recommendations.

#### Left ventricular failure

**Fig. 1:**
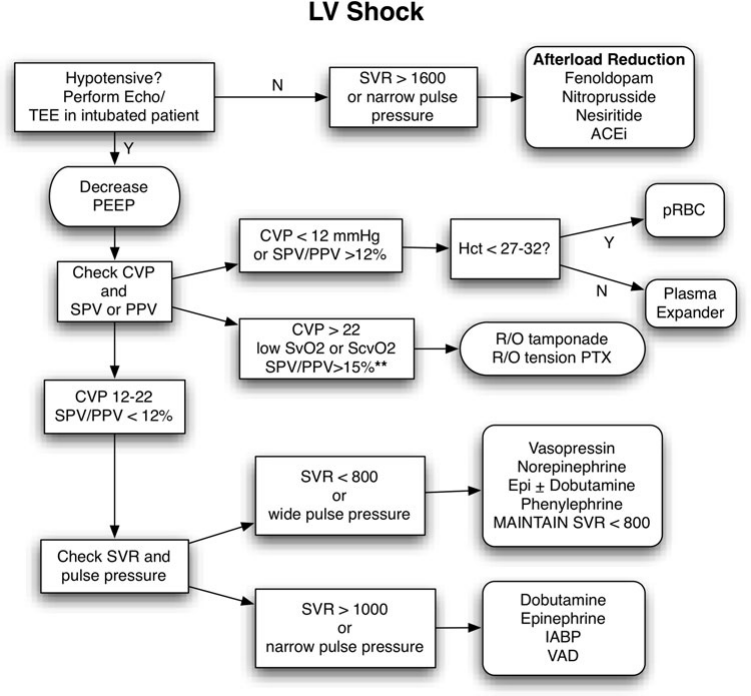
Treatment algorithm of left ventricular failure with cardiogenic shock. SPV = systolic pressure variation; SVR = systemic vascular resistance in dyne·sec^−1^·cm^5^·m^2^; PTX = pneumothorax; IABP = intraortic balloon pump; VAD = ventricular assist device; PEEP = positive end-expiratory pressure; ACEi = angiotensin-converting enzyme inhibitor

The management of left ventricular (LV) failure is substantially different from the management of RV failure. In both settings, an adequate circulating volume is essential for ventricular filling and forward flow. The failing LV is best supported with afterload reduction when possible, followed by the administration of positive inotropic agents.^71^,^72^ Mechanical assist devices are available in some settings to support the patient with LV failure, but escalation of therapy to that level may not always be possible or clinically appropriate (Fig. 1).

#### Right ventricular failure

**Fig. 2:**
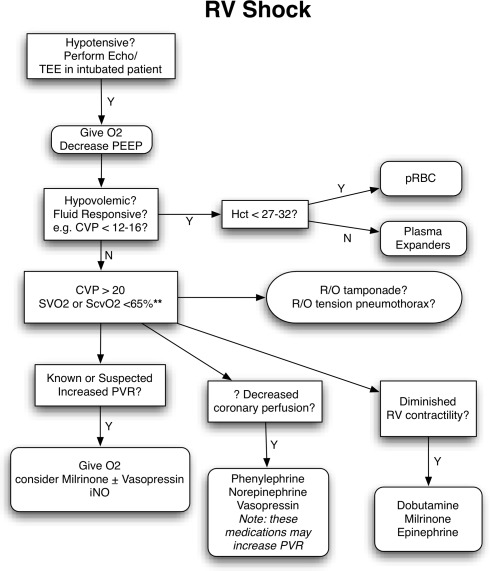
Treatment algorithm of right ventricular failure with cardiogenic shock. iNO = inhaled nitric oxide

The failing RV is best managed with a combination of pulmonary vasodilators and positive inotropic agents. Unlike the setting of LV failure that may require afterload reduction, the use of systemic arterial vasoconstrictors for RV dysfunction may improve end-organ perfusion and cardiac output (Fig. 2).^73^,^74^ At present, mechanical assist devices are not used to manage the vast majority of patients with RV failure. With the exception of patients with infarction of the RV, the following most common causes of an RV-limited circulation share the pathophysiology of elevated pulmonary vascular resistance: severe obstructive lung disease (including chronic obstructive pulmonary disease [COPD] and chronic bronchitis), obstructive sleep apnea with sleep-disordered breathing, morbid obesity, massive pulmonary embolism, recurrent thromboembolism, primary pulmonary hypertension, and pulmonary hypertension from inflammatory destruction of pulmonary capillaries (e.g., systemic sclerosis).^73^

#### Clinical progression to shock

Some patients will continue to deteriorate despite volume infusions. In these cases, anesthesiologists may administer small boluses of vasopressor drugs or initiate inotropic support.

If therapy with catecholamines does not improve hemodynamics, small boluses of vasopressin (arginine vasopressin [AVP] 0.5-2 U *iv*) may work where other catecholamines have failed. Extensive literature documents the use of AVP and its analogs in low-flow states, cardiopulmonary arrest, and general and regional anesthesia.^75^-^78^ Given the autonomic imbalance of the perioperative period, consideration should be given to the use of atropine in any patient who does not manifest the anticipated tachycardic response in a crisis.^79^

If the patient’s clinical condition continues to deteriorate, several immediate diagnostic and corrective measures should be considered, including focused echocardiography and, if feasible, reduction of anesthetics (Table 7).^80^-^82^

**Table 7:** Corrective measures for clinical progression to shock and a modified stepwise approach for cardiac arrest in the operating room based on the American Heart Association’s 2010 ACLS Guidelines and the 2008 International Liaison Committee on Resuscitation Consensus Statement on post-cardiac arrest syndrome

**Corrective Measures for Clinical Progression to Shock**	
• Recognize a true crisis	
• Call for help	
• Call for defibrillator	
• Hold surgery and anesthetic if feasible	
• Administer FIO_2_ of 1.0	
• Confirm airway positioning and functioning	
• Assess oxygen source and anesthetic circuit integrity	
• Review E_T_CO_2_ trends before hemodynamic instability	
**Generate a Differential Diagnosis**	
• Evaluate procedure and consult with procedural colleagues	
• Review recently administered medications	
• Obtain chest radiograph to rule out tension pneumothorax if airway resistance acutely increased	
• Obtain echocardiogram (transesophageal echocardiogram if patient’s trachea is intubated or if patient has a surgically prepped chest) to evaluate ventricular filling, ventricular function, and valvular function, and to exclude pericardial tamponade (Focused Echocardiographic Evaluation and Resuscitation [FEER] exam^80^)	
• Empiric replacement therapy with corticosteroids (in patients who have not been previously treated with steroids, hydrocortisone 50 mg *iv* and fludrocortisone 50 μg *po*/nasogastric is an appropriate dose^81^,^82^)	
**Perioperative Cardiac Arrest**	
Circulation	• Check pulse for 10 sec
	• Effective two-rescuer CPR:
	1. Minimize interruptions
	2. Chest compression rate 100 compressions·min^−1^
	3. Depth 2 in, full decompression, real-time feedback.
	4. Titrate CPR to A-line BP diastolic 40 mmHg or E_T_CO_2_ 20 mmHg
	• Drug Rx
	• Attempt CVL placement
Airway	• Bag mask ventilation until intubation
	• Endotracheal intubation
	• Difficult airway algorithm
Breathing	• Respiratory rate 10 breaths·min^−1^
	• V_T_ to visible chest rise
	• T_I_ 1 sec
	• Consider inspiratory threshold valve (ITV)
Defibrillation	• Defibrillation if shockable rhythm
	• Repeat defibrillation every 2 min if shockable rhythm
**Post Cardiac Arrest**	
• Invasive monitoring	
• Final surgical anesthetic plan	
• Transfer to ICU	

ACLS = advanced cardiac life support; BLS = basic life support; CPR = cardiopulmonary resuscitation; FIO_2_ = fraction of inspired oxygen concentration; E_T_CO_2_ = end-tidal carbon dioxide; BP = blood pressure; CVL = central venous line; V_T_ = tidal volume; T_I_ = inspiratory time; ICU = intensive care unit

#### Ventilation during severe shock or cardiac arrest

Practitioners may be concerned that reducing ventilation in a patient in shock or in severe respiratory failure will have deleterious effects. Although this has been the conventional wisdom in anesthesia and medicine, studies from the past 15 years suggest a survival benefit from moderate hypoventilation and respiratory acidosis. For example, hypoventilation is associated with a lower incidence of barotrauma in patients with acute respiratory distress syndrome (ARDS) or COPD and is rarely a cause of hypoxemia. Patients with severe lung disease can tolerate hypercarbia and respiratory acidosis.^83^-^88^

Hyperventilation is deleterious in all conditions of low-flow state. Studies of ventilation during shock emphasize the following principle: In a low-flow state, the duration of increased intrathoracic pressure is proportional to the ventilation rate, tidal volume, inspiratory time, and delayed chest decompression and is inversely proportional to coronary and cerebral artery perfusion.^84^,^89^-^91^ For example, ventilation at 20 breaths·min^−1^ during CPR is associated with significantly lower survival than ventilation at 10 breaths·min^−1^. The most recent ACLS guidelines emphasize avoiding hyperventilation during CPR until an advanced airway device (endotracheal tube, laryngeal mask airway device, or esophageal airway) is inserted. This can be achieved via a higher compression:ventilation ratio (30:2) for single-rescuer CPR for victims of all ages (except newborns) and for two-rescuer CPR for adult victims. Once an advanced airway is in place, the respiratory rate should be maintained at no more than 10 breaths·min^−1^ with an inspiratory time of one second and the tidal volume limited to “chest rise” (approximately 500 mL in an average 70 kg adult).^3^ Capnography is usually a more reliable indicator of ROSC than carotid or femoral arterial pulse palpation.^3^ If rescuers are concerned that the capnograph is malfunctioning, blowing into the sidestream CO_2_ collecting tube is a quick way to assess this.

New technologies and devices providing automatic CPR^92^,^93^ and an airway in-line negative inspiratory valve providing increased venous return during chest decompression^94^ have all been associated with an increased rate of ROSC, although no clear increase in hospital discharges has been observed.

#### Auto-positive end-expiratory pressure

Auto-positive end-expiratory pressure (auto-PEEP), also known as *intrinsic PEEP* or *gas trapping*, is a well-described cause of circulatory collapse and may be difficult to recognize as a cause of PEA/electromechanical dissociation.^95^ It occurs almost exclusively in patients with obstructive lung disease, including asthma and COPD (emphysema), and is exacerbated by hyperventilation. In these patients, patterns of ventilation that do not allow sufficient time for complete exhalation produce a gradual increase in the end-expiratory volume and pressure in the lung. This pressure is transmitted to the great veins in the thorax, which depresses both venous return and cardiac output. As auto-PEEP increases, venous return declines.^96^,^97^ Auto-positive end-expiratory pressure should be considered a cause of rapid hemodynamic deterioration in an anesthetized patient with acute bronchospasm.

**Fig. 3:**
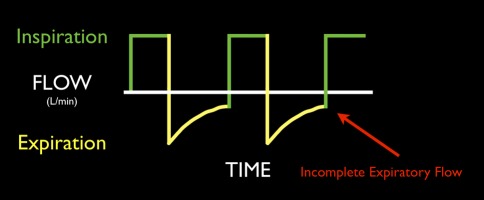
Auto-PEEP during mechanical ventilation. Failure of the expiratory waveforms to return to zero baseline before the next inspiration is indicative of the presence of auto-PEEP. PEEP = positive end-expiratory pressure

Detecting and decreasing auto-PEEP is a straightforward way to support a sagging circulation. In Fig. 3, the failure of the expiratory flow waveforms to return to zero baseline before the next inspiration is indicative of the presence of auto-PEEP. In the absence of a flow waveform display, auto-PEEP should be ruled out by temporarily disconnecting the endotracheal tube and observing the “step-up” gain of invasive or noninvasive systolic blood pressure. A ROSC upon discontinuance of all resuscitative maneuvers, described as the *Lazarus phenomenon*, is most likely the consequence of sudden termination of extreme dynamic hyperinflation during CPR, particularly when hyperventilation is applied.^98^

If auto-PEEP is suspected as the cause of circulatory crisis, disconnecting a patient’s tracheal tube from the ventilator for a brief time (5-10 sec) can produce a dramatic improvement in the circulation. Patients who demonstrate dramatic improvement in response to this maneuver will benefit from maximal therapy for obstruction/bronchospasm and will likely fare best with a reduced respiratory rate, short inspiratory time (to promote a longer expiratory time), and lower tidal volumes (no more than 8 mL·kg^−1^).

#### Hypovolemia and systolic and pulse pressure variation

**Fig. 4:**
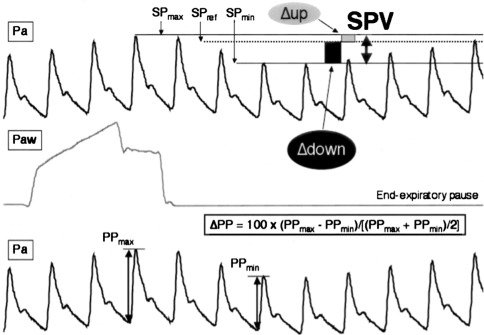
Analytical description of respiratory changes in arterial pressure during mechanical ventilation. The systolic pressure and the pulse pressure (systolic minus diastolic pressure) are maximum (SPmax and PPmax, respectively) during inspiration and minimum (SPmin and PPmin, respectively) a few heartbeats later, i.e., during the expiratory period. The systolic pressure variation (SPV) is the difference between SPmax and SPmin. The assessment of a reference systolic pressure (SPref) during an end-expiratory pause allows the discrimination between the inspiratory increase (Δup) and the expiratory decrease (Δdown) in systolic pressure. Pa = arterial pressure; Paw = airway pressure. (This figure is reproduced with permission from the publisher. *Michard F:* Changes in arterial pressure during mechanical ventilation. Anesthesiology 2005; 103: 419-28)

Hypovolemia is a common cause of perioperative hypotension. Reliable indicators of fluid responsiveness (an increase in stroke volume with fluid administration) in patients receiving tidal volumes of at least 8 mL·kg^−1^ include systolic and pulse pressure variation and stroke volume variation.^99^,^100^ The greater the fluctuation of the systolic pressure and pulse pressure of an arterial catheter tracing with mechanical ventilation, the more likely the patient will respond (increase cardiac output) to volume infusion (Fig. 4). Plethmysography may show a similar large respiratory variation.^101^-^103^ Unfortunately, the pulsatile part of the signal is diminished in the presence of low cardiac output or increased systemic vascular resistance.^104^ Elevations in pulse pressure and systolic pressure variation may not always suggest fluid responsiveness in a few but important clinical circumstances, including RV shock and all causes of obstructive shock (e.g., auto-PEEP, cardiac tamponade, tension pneumothorax, pulmonary hypertension, and abdominal compartment syndrome).^90^,^105^

The corollary is also true: Minimal or absent systolic and pulse pressure variation with respiration strongly suggests that interventions, other than the infusion of volume or increasing venous return via vasopressors, will be required to support the circulation (e.g., adding positive inotrope agents or eliminating negative inotrope agents). Importantly, excessive tidal volumes (>10 mL·kg^−1^), increased residual volume and lung compliance (emphysema), and decreased chest wall compliance (3rd degree chest burn, obesity, prone position) will also cause an increase in systolic pressure variation requiring practitioners to make incremental adjustments to their criteria for volume responsiveness.^99^ Additional indices of fluid responsiveness include the passive leg raise (a quick, reversible, and easy to perform maneuver in which the patient’s legs are raised and a change in blood pressure is assessed—useful in spontaneously ventilating patients), variation in inferior vena cava diameter with respiration via ultrasonography (if access to the abdomen is possible), and esophageal Doppler assessment of aortic velocity.^100^,^106^-^112^

In the setting of severe hypotension, it is reasonable for practitioners to provide volume resuscitation, as long as there is an increase in blood pressure and/or cardiac output without an increased requirement of FIO_2_ or worsening of total lung compliance (both possible signs of cardiogenic pulmonary edema).

#### Rescue sequence for cardiac arrest in the operating room

For a variety of reasons, recognizing the time to commence CPR in the OR is more difficult than it may appear to outsiders. First, false alarms vastly outnumber real events because disconnection of sensors (“asystole”), blood draws, and electrocautery are more common than cardiac arrest in most ORs.^113^,^114^ Monitoring devices can also fail from heavy use. Second, hypotension and bradycardia are relatively common occurrences in the OR, and most patients recover to an adequate hemodynamic status with minimal intervention. Third, it can be difficult or impossible to obtain satisfactory monitoring in many patients, especially those with vasculopathy, hypothermia, burns, vasoconstriction, or morbid obesity.^115^

The features of cardiac arrest in the OR include ECG with pulseless rhythm (ventricular tachycardia [V-tach], ventricular fibrillation [V-fib], severe bradycardia, and asystole), loss of carotid pulse > ten seconds, loss of end-tidal CO_2_ with loss of plethysmograph, and/or loss of arterial line tracing. Once cardiac arrest is confirmed, effective CPR should be initiated immediately in the OR. More important, the rescuer should provide physiological feedback and monitor the data from the ECG, pulse oximetry, end-tidal CO_2_ (E_T_CO_2_), CVP, and arterial catheter. Effective chest compression is indicated by an E_T_CO_2_ close to or above 20 mmHg. In 100% of cases, an E_T_CO_2_ <10 mmHg after 20 min of standard ACLS is associated with failure of ROSC.^116^-^119^ A relaxation (diastolic) pressure (calculated at the time of full chest decompression) of 30-40 mmHg in the presence of an arterial catheter has been associated with a higher rate of ROSC, even after prolonged CPR.^120^-^122^ Feedback on the quality of chest compressions can be provided by some of the new defibrillators.^123^

In the presence of a CVP, the estimate of coronary perfusion pressure (CPP) during CPR is more accurate than by aortic relaxation only. Coronary perfusion pressure can be estimated by briefly freezing the monitoring screen with the arterial line and synchronizing the CVP waveforms. Coronary perfusion pressure is calculated at the time of full chest decompression and is equal to the arterial catheter pressure minus CVP. A CPP > 15 mmHg is associated with an increased rate of ROSC.^124^,^125^

**Fig. 5:**
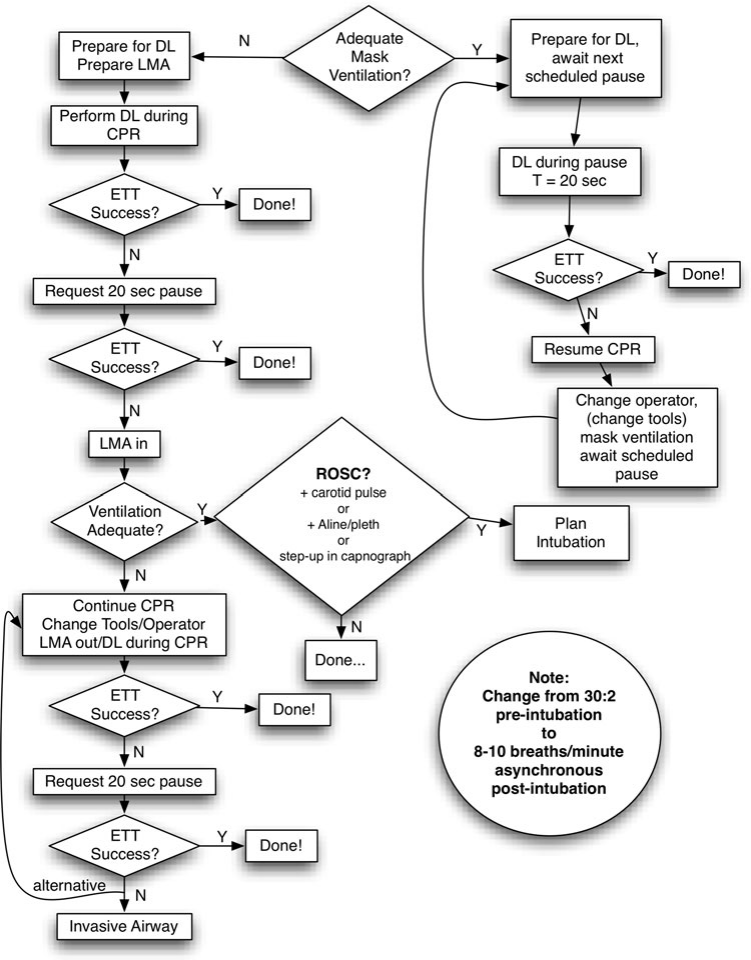
Intubation. Algorithm for managing the airway and producing the least possible disruption in chest compressions DL = direct laryngoscopy; LMAD = laryngeal mask airway device; ETT = endotracheal tube

Table 7 shows a stepwise therapeutic approach to cardiac arrest in the OR which is based on the 2010 American Heart Association ACLS sequence and the International Liaison Committee on Resuscitation consensus statement on post-cardiac arrest syndrome.^126^ An algorithm for coordinating airway management with CPR is shown in Fig. 5.

### ACLS operating room algorithms

#### Symptomatic bradycardia evolving to nonshockable arrest

Sixteen causes (8H and 8T) of perioperative bradycardia and nonshockable cardiac arrest (asystole and PEA) are listed in Table 8 as an extension of the current “6H and 6T”-based American Heart Association algorithm.^3^ Bradycardia and hypotension caused or precipitated by vagotonic analgesics, physical manipulations that increase vagal tone, and sympatholysis from anesthetic agents and regional/neuraxial anesthetic techniques require immediate evaluation and intervention.

**Table 8:** Amnemonic approach to the differential diagnosis of bradycardia and nonshockable cardiac arrest

Hypoxia	Toxins (anaphylaxis/anesthesia)
Hypovolemia	Tension pneumothorax
Hyper-/Hypokalemia	Thrombosis/Embolus, pulmonary
Hydrogen ion (acidemia)	Thrombosis coronary
Hypothermia	Tamponade
Hypoglycemia	Trauma (hemorrhagic shock, CV injury)
Malignant Hyperthermia	qT prolongation
Hypervagal	Pulmonary hyperTension

CV = cardiovascular

**Fig. 6:**
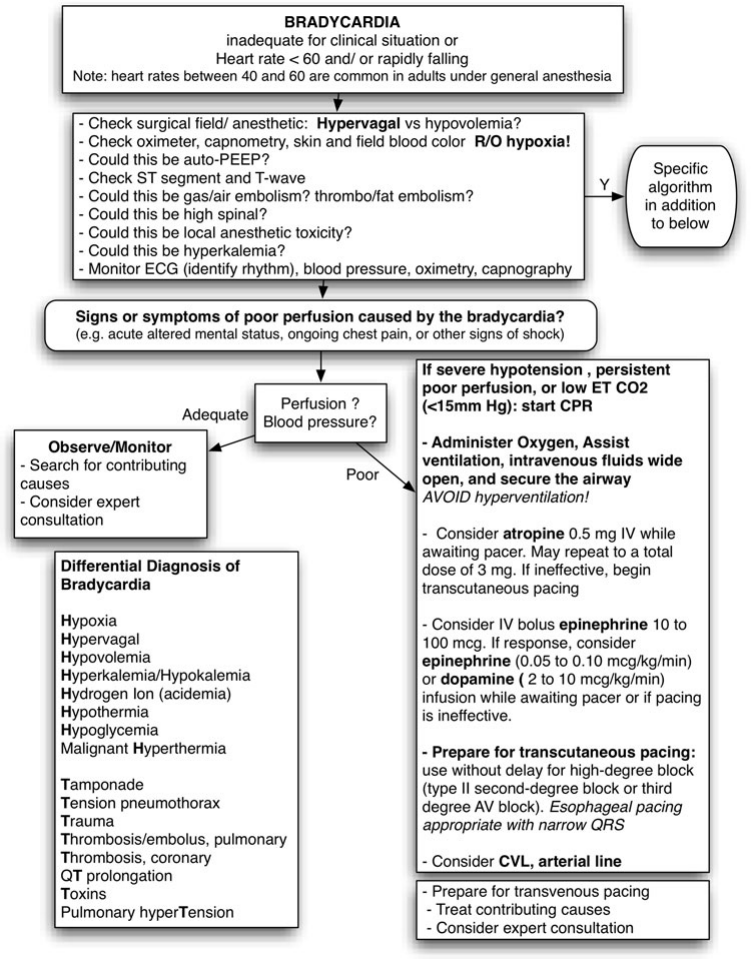
Bradycardia. With information from the ACLS algorithm for bradycardia. ACLS = advanced cardiac life support; CVL = central venous line or catheter; E_T_CO_2_ = end-tidal carbon dioxide

The different spectrum of causes of bradycardia makes it reasonable to attempt earlier pacing in this patient population than in most other settings. Failure to intervene in a timely fashion can allow the patient to deteriorate into a nonshockable cardiac arrest. However, there is no evidence to suggest any advantage from the use of pacing (which may delay chest compressions) when full cardiac arrest is in progress.^127^-^130^ Typical indications for emergency pacing include hemodynamically symptomatic bradycardia unresponsive to positive chronotropic agents, regardless of the cause; symptomatic tissue conduction dysfunction of the sinus node; Mobitz type II second degree and third degree block; alternating bundle branch block; or bifascicular block.^3^ A treatment sequence approach to perioperative bradycardia is given in Fig. 6.

#### Symptomatic tachycardia evolving to pulseless shockable arrest (ventricular tachycardia, ventricular fibrillation, and torsades de pointes)

Although symptomatic tachycardia, often triggered by severe hypovolemia or an imbalance between surgical stimulus and anesthetic depth, is frequent in the perioperative arena, data regarding its incidence and management are lacking.^131^ The differential diagnosis for nonshockable tachycardia includes the 8Hs and 8Ts.

Evolution to a malignant rhythm is unlikely in the absence of severe cardiac comorbidities and/or anesthesia complications. Persistent hemodynamic instability with tachycardia can sometimes degenerate rapidly to symptomatic bradycardia. Immediate cardioversion is indicated for a patient with hemodynamic instability from tachycardia (ventricular rate > 150 beats·min^−1^).^3^ Cardiac pacing may be necessary in patients after cardioversion because some patients’ rhythms will convert to symptomatic bradycardia. Overdrive pacing of supraventricular or ventricular tachycardia refractory to drugs or electrical cardioversion is also appropriate in perioperative patients.^132^

**Fig. 7:**
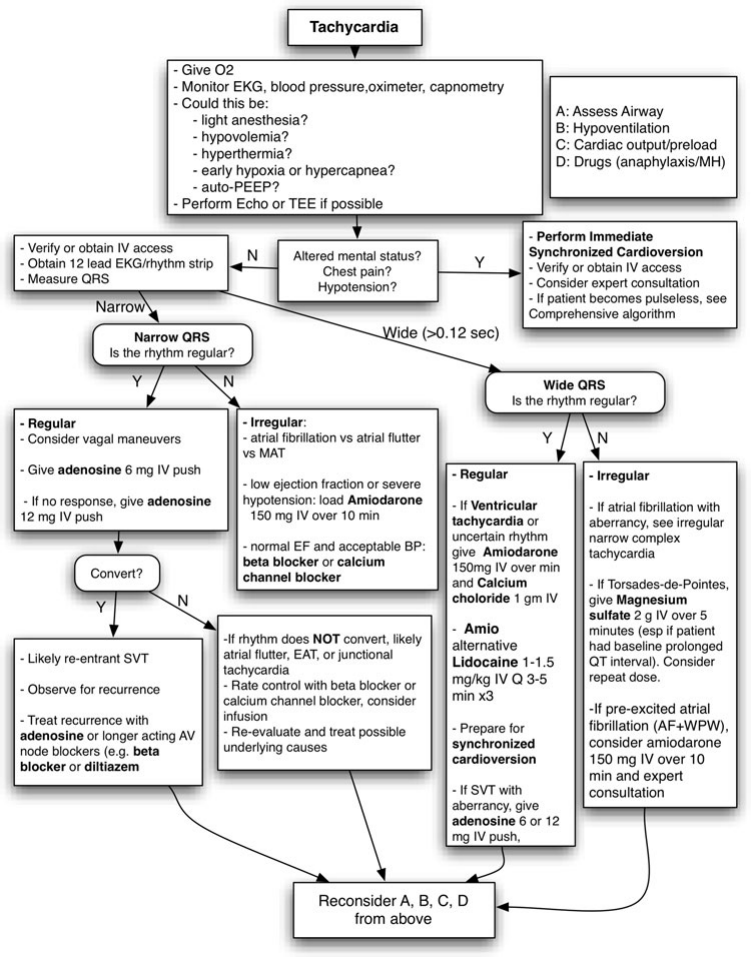
Tachycardia. With information from the ACLS algorithm for tachycardia. ACLS = advanced cardiac life support; TEE = transesophageal echocardiography; MH = malignant hyperthermia; EF = ejection fraction; MAT = multifocal atrial tachycardia; EAT = ectopic atrial tachycardia; SVT = supraventricular tachycardia; AF + WPW = atrial fibrillation and Wolff-Parkinson-White syndrome

**Fig. 8:**
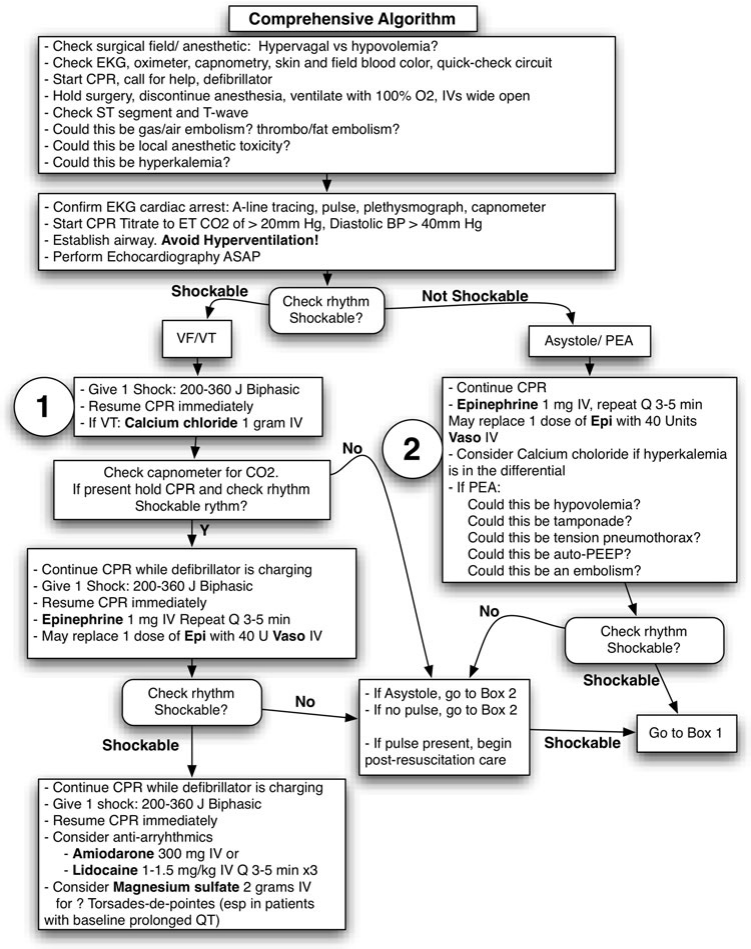
Comprehensive algorithm. With information from the ACLS comprehensive algorithm. Rescuers are prompted to evaluate or empirically treat early for hyperkalemia. Echocardiography is especially useful in establishing the most likely cause of pulseless electrical activity and focusing resuscitation efforts. ACLS = advanced cardiac life support

In Table 9 and Figs. 7 and 8, there are lists of practical considerations for the management of symptomatic tachycardia in the perioperative period.

**Table 9:** Pharmacological approach to shockable rhythm post defibrillation and recommended infusion doses

Rhythm	1st Line	2nd Line	3rd Line
Ventricular tachycardia	*Amiodarone	^†^Lidocaine	^‡^Procainamide
Ventricular fibrillation	Amiodarone		
Torsades de pointes	^‖^Magnesium		

* Amiodarone: 300 mg *iv* bolus, then 150 mg *iv* bolus. Repeat every 5 min up to 1.5 g total. Infusion rate: 1 mg·min^−1^

^†^Lidocaine: Load 1.0-1.5 mg·kg^−1^ bolus. Repeat 0.5-0.75 mg·kg^−1^ 3-5 min later. Maximum dose 3 mg·kg^−1^. Infusion range: 1-4 mg·min^−1^

^‡^Procainamide: 20-50 mg·min^−1^ *iv*. Maximum: 17 mg·kg^−1^. Infusion rate: 1-4 mg·min^−1^

^‖^Magnesium sulfate: 2 g *iv* for torsades de pointes, hypomagnesemia, or hypokalemia. May repeat three times

### Conclusion

Cardiac arrest in the perioperative setting is rare and has a different spectrum of causes that compel situation-specific adaptations of ACLS algorithms. In fact, there are intuitive differences in patient management when the health care provider has prior knowledge of a patient’s medical history, is immediately aware of the probable cause of arrest, and initiates medical management within seconds. Since it is both uncommon and heterogeneous, perioperative cardiac arrest has not been described or studied to the same extent as cardiac arrest in the community; thus, recommendations for management must be predicated on expert opinion and physiological understanding rather than on the standards currently being used in the generation of ACLS protocols in the community.
